# KRASAVA—An Expert System for Virtual Screening of KRAS G12D Inhibitors

**DOI:** 10.3390/ijms27010120

**Published:** 2025-12-22

**Authors:** Oleg V. Tinkov, Pavel E. Gurevich, Sergei A. Nikolenko, Shamil D. Kadyrov, Natalya S. Bogatyreva, Veniamin Y. Grigorev, Dmitry N. Ivankov, Marina A. Pak

**Affiliations:** 1 Ligand Pro, Moscow 121205, Russiad.ivankov@skoltech.ru (D.N.I.);; 2 Artificial Intelligence Center, Moscow 121205, Russia; 3 Institute of Protein Research, Russian Academy of Sciences, Pushchino 142290, Russia; 4 Institute of Physiologically Active Compounds, Federal Research Center of Problems of Chemical Physics and Medicinal Chemistry, Russian Academy of Sciences, Chernogolovka 142432, Russia; 5 Center for Molecular and Cellular Biology, Moscow 121205, Russia

**Keywords:** QSAR, machine learning, structural interpretation, RDKit, molecular docking

## Abstract

The development of KRAS G12D inhibitors represents an effective therapeutic strategy for treating oncological pathologies. Existing quantitative structure-activity relationship (QSAR) models for KRAS G12D inhibitors have several limitations, primarily the lack of applicability domain determination and virtual screening implementation. In this study, we propose a set of regression QSAR models for KRAS G12D inhibitors by employing various molecular descriptors and machine learning methods. Our consensus model achieved a *Q*^2^ test value of 0.70 on an external test set, covering 78% of the data within the applicability domain. We integrated this consensus model into our Python-based framework KRASAVA. The platform predicts inhibitory activity while considering the applicability domain, assesses compounds for compliance with Muegge’s bioavailability rules, and identifies PAINS, toxicophores, and Brenk filters. Furthermore, we structurally interpreted the QSAR models to propose several promising inhibitors and performed molecular docking on these candidates using GNINA. For the reference inhibitor MRTX1133, we reproduced the crystal structure pose with an RMSD of 0.76 Å (PDB ID: 7T47). The key interactions with amino acid residues Asp12, Asp69, His95, Arg68, and Gly60, identified for both MRTX1133 and our proposed compounds, demonstrate a strong consistency between the molecular docking and QSAR results.

## 1. Introduction

The KRAS gene (Kirsten rat sarcoma viral oncogene) encodes a protein from the Ras GTPase family playing a central role in cellular signaling by regulating proliferation, differentiation, and cell survival [1,2]. Normally, the KRAS protein cycles between an active (GTP-bound) and inactive (GDP-bound) state. The G12D mutation, involving the mutation of glycine with aspartic acid at position 12, is among the most common and aggressive KRAS mutations [3,4,5,6]. The G12D mutation constitutively activates KRAS, directly causing uncontrolled cell proliferation, tumor formation, and alterations in the tumor microenvironment. This direct causal link makes KRAS G12D a critically important oncological target, whose inhibition can fundamentally alter disease progression [7]. The G12D mutation locks KRAS in the active state, leading to continuous stimulation of downstream signaling pathways such as MAPK/ERK and PI3K/AKT. Persistent activation of these pathways drives uncontrolled cell growth, tumor formation, and modification of the tumor microenvironment [8]. The G12D mutation is the most common subtype of KRAS mutations in pancreatic cancer and a frequently occurring dominant subtype in colorectal cancer [9]. Consequently, G12D is not merely a marker but an active driver of cancer pathogenesis. Targeting this mutation addresses the core mechanism of oncogenesis, explaining its importance in drug development [10]. Recent progress in the development of KRAS G12D inhibitors, including MRTX1133 and ASP3082, demonstrate the clinical relevance of targeting the G12D mutation [11,12].

In modern pharmacology, drug discovery remains a complex and resource-intensive process. Quantitative structure–activity relationships (QSAR) serve as a fundamental statistical tool to correlate the chemical structure of molecules with their biological activity through molecular descriptors [13]. QSAR offers significant value by predicting the biological activity of novel compounds, thereby reducing costly and labor-intensive experimental assays and accelerating the drug discovery timeline. QSAR models play a pivotal role in the early stages of drug development, enabling the efficient elimination of compounds with undesirable properties prior to extensive laboratory testing [14]. A critical aspect of drug development is the prediction and understanding of toxicity and bioavailability profiles, as toxicity causes preclinical failure for approximately 30% of compounds [15], and inadequate pharmacokinetics eliminates up to 15% of candidates at the preclinical stage [16].

For example, MRTX1133 is a highly selective, non-covalent inhibitor of the mutant KRAS G12D protein. Preclinical studies demonstrated its exceptional potency and specificity (IC_50_ < 2 nM, ~700–1000-fold selectivity over KRAS WT) and induced significant tumor regression in xenograft and immunocompetent mouse models [17,18]. Despite the promise of MRTX1133 as a KRAS G12D inhibitor, preclinical pharmacokinetic studies revealed challenges for its therapeutic development. A 2024 study [19] in rats reported very low oral bioavailability of MRTX1133 at only 2.92% and a short plasma half-life of 1.12 h after oral administration. These data indicated potential difficulties in achieving and maintaining adequate therapeutic concentrations in humans, a critical factor for successful clinical translation. Clinical trials of MRTX1133 (phase 1/2), initiated in March 2023, were prematurely terminated in early 2025 after phase 1 completion. The reasons included unstable and inadequate pharmacokinetics alongside highly variable and unsatisfactory bioavailability data [20].

This example highlights the necessity of thoroughly evaluating both inhibitory activity and ADMET properties for KRAS G12D inhibitors. Traditional oral bioavailability evaluation relies on rules introduced by Lipinski [21], Muegge [22], Ghose [23], Veber [24], and Egan [25]. Among these, Muegge’s rules incorporate the largest set of parameters for bioavailability assessment, making them especially useful at early development stages. Muegge’s rules define an expanded drug-likeness filter that sets explicit thresholds—molecular weight between 200 and 600 Da, logP ≤ 5, ≤10 hydrogen-bond acceptors, ≤5 hydrogen-bond donors, ≤15 rotatable bonds, TPSA ≤ 150 Å^2^, and ≤7 rings—to rigorously prioritize bioavailability compounds during early-stage virtual screening. Notably, MRTX1133 does not comply with Muegge’s criteria, showing a molecular weight exceeding 600 Da and possessing more than seven ring systems. Various medicinal chemistry filters are applied early in drug development to assess compound compliance with established bioavailability rules and to exclude structural alerts such as Brenk filters and PAINS. Brenk filters consist of 105 structural fragments that increase toxicity risk, impair pharmacokinetic properties, and generally reduce suitability for drug candidates [26]. Pan-assay interference compounds (PAINS) represent chemical entities that frequently produce false-positive results in high-throughput screening. PAINS exhibit nonspecific interactions across multiple biological targets rather than selectively acting on the intended target [27].

Given the relevance of KRAS inhibition as a therapeutic strategy, several research groups have developed satisfactory QSAR models linking chemical structure to inhibitory activity against KRAS [28,29,30,31]. Srisongkram et al. constructed robust QSAR models using a dataset of 1033 compounds, achieving $Q_{cv}^{2}$ = 0.60 and $Q_{ext}^{2}$ = 0.62 [28]. Srisongkram and Weerapreeyakul [29] proposed both classification and regression QSAR models targeting drug repurposing of FDA-approved compounds as KRAS G12C inhibitors, using a dataset of 1255 molecules with reported metrics of $Q_{cv}^{2}$ = 0.60, $Q_{ext}^{2}$ = 0.62, Accuracy _cv_ = 0.84, Accuracy _ext_ = 0.85. Both studies [28,29] employed compounds with experimentally measured IC_50_ values against KRAS G12C and applied the XGBoost gradient boosting algorithm together with molecular descriptors, including PubChem and substructural fingerprints. The authors utilized SHAP-based interpretation to identify key fragment contributions. The QSAR results from both studies received further validation through molecular docking.

Studies [30,31] proposed classification-based QSAR models for KRAS G12D inhibitors. Despite acceptable statistical performance, these models present a major limitation: they lack an explicitly defined applicability domain, violating a key QSAR modeling principle established by the OECD expert group [32]. Without this domain, QSAR models cannot meet regulatory suitability criteria. Additionally, these studies [30,31] do not clearly detail data curation and preprocessing procedures, a critical step given the risk of errors and inconsistencies in publicly available chemical databases and other sources [33]. To identify the most active compounds during virtual screening, it is advisable to employ regression models that predict the inhibitory activity levels in greater detail than classification models.

Importantly, the effective application of QSAR models for KRAS inhibitors in medicinal chemistry requires appropriate software tools, either desktop or web-based. Unfortunately, the aforementioned studies [28,29,30,31] did not provide such tools.

In this study, we aimed to construct regression QSAR models for KRAS G12D inhibitors using various molecular descriptors and machine learning methods and to integrate the developed QSAR model into our web-based Python v3.11 framework, KRASAVA (KRAS Automated Virtual Assistant), enabling virtual screening of KRAS G12D inhibitors with simultaneous bioavailability assessment based on Muegge’s rules.

**Figure 1:**
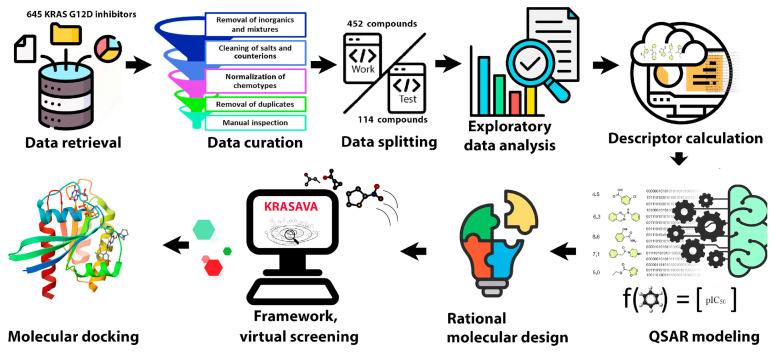
General workflow of the study.

The main stages of this study are presented in Figure 1: (1) collection of experimental data of KRAS G12D inhibitors; (2) data validation taking into account generally accepted recommendations [33]; (3) division of the total dataset into training and testing sets; (4) exploratory data analysis; (5) calculation of molecular descriptors; (6) development, validation and structural interpretation of QSAR regression models; (7) rational molecular design; (8) development of the KRASAVA framework as a reproducible Jupyter Notebook v7.5., executable via Google Colab with subsequent implementation of the best QSAR models; (9) molecular docking of the promising compounds under study. A detailed description of each stage is provided in the Methods and Materials section.

## 2. Results and Discussion

We developed several QSAR models for KRAS G12D inhibitors as described in Materials and Methods. Table 1 contains the statistical parameters of the QSAR models. All developed models of KRAS G12D inhibitors exhibit satisfactory statistical characteristics and demonstrate comparable predictive performance. The models developed using ECFP4, Topological Path-Based fingerprints, and 2D RDKit descriptors and the CatBoost algorithm demonstrated the best statistical performances (Table 1, the QSAR models highlighted in bold). The *p*-values for these models under y-randomization are below 0.02, confirming the absence of random correlations in the proposed QSAR models. The consensus model was developed by integrating the above three best models. The applicability domain in consensus forecasting is calculated using ECFP4 fingerprints, since they have the smallest data coverage compared to other types of descriptors used in the consensus model.

The predictive ability of the consensus QSAR model was further validated using a second independent external test set. Due to the relatively small structural space described by the proposed QSAR model, only 21 compounds were included in the applicability domain, with a Q^2^_ts_ of 0.73, demonstrating a sufficiently high level of predictive ability for the proposed consensus QSAR model.

Additionally, we developed 63 QSAR models using the OCHEM platform; see their statistical parameters in Appendix A, Table A1. We obtained the best results by the Random Forest method using, again, ECFP4 descriptors: $Q_{cv}^{2}$ = 0.66; $Q_{ts}^{2}$ = 0.69. The model is publicly available at https://ochem.eu/model/20748154 (accessed on 10 December 2025).

**Figure 2:**
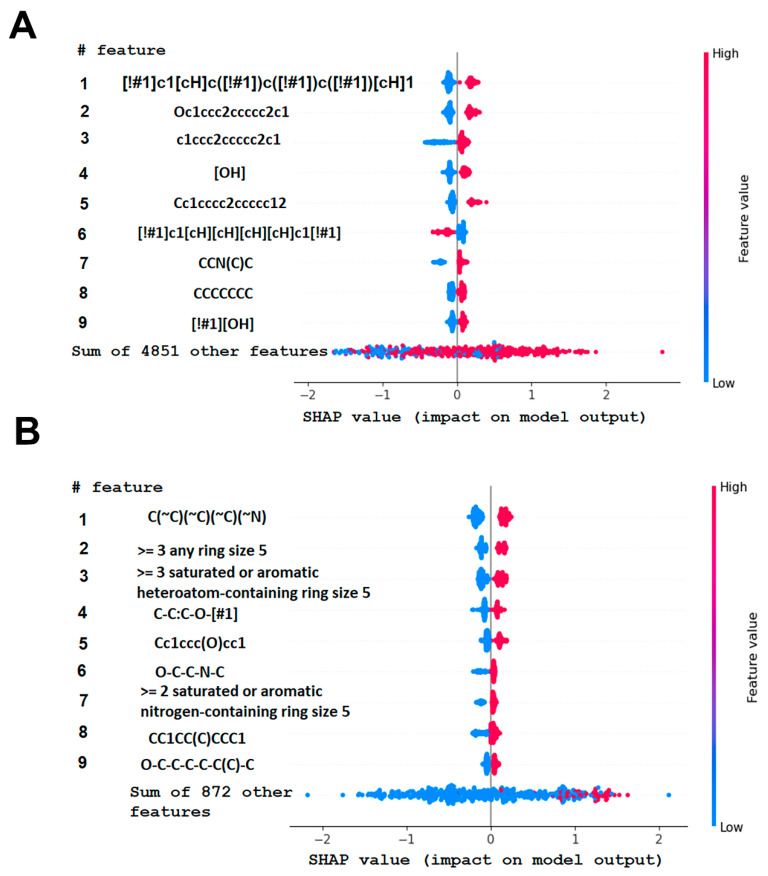
Results of the structural interpretation of QSAR models of KRAS inhibitors developed using Klekota-Roth (**A**) and PubChem (**B**) descriptors. Symbol # denotes the number of feature.

To investigate the influence of structural features on the inhibitory activity of KRAS inhibitors, we performed a structural interpretation of the QSAR models developed using Klekota-Roth and PubChem descriptors (Figure 2 and Table A2 and Table A3). In addition, we carried out a matched molecular pair analysis (MMPA) for the best QSAR model developed using the OCHEM platform (Table 2).

Summarizing the obtained results, we can identify the following main structural modifications that increase the inhibitory activity of KRAS G12D inhibitors:

1. Substitution of a nitrile group with a hydroxy or alkyne group (molecular transformations 1 and 5 in Table 2), which is consistent with the high contribution to activity of descriptors No. 4 and 9 shown in Figure 2A and Table A2, as well as descriptors No. 4, 6, and 9 shown in Figure 2B and Table A3;
2. Replacement of a methoxy group with an alkyne group (molecular transformation 2 in Table 2);
3. Replacement of a pyridine fragment with a 1-methylpyrrolidine fragment (molecular transformation 3 in Table 2);
4. Substitution of a methoxy group with a hydroxy group (molecular transformation 4 in Table 2);
5. Replacement of an ethylene fragment with a pyrrolizidine fragment (molecular transformation 6 in Table 2);
6. Introduction of a hydroxyl group in the para-position (molecular transformation 7 in Table 2), which correlates with the high contribution to activity of descriptors No. 4 and 9 (Figure 2A, Table A2) and descriptors No. 4, 6, and 9 (Figure 2B, Table A3);
7. Elongation of the linker connected to the imidazole ring (molecular transformation 8 in Table 2);
8. Transformation of a phenyl group into a naphthyl group, consistent with the high contribution to activity of descriptors No. 2, 3, and 5 (Figure 2A, Table A2) (molecular transformation 9 in Table 2);
9. Replacement of a pyridine fragment with an imidazole ring (molecular transformation 10 in Table 2).

We integrated the consensus QSAR model into the KRASAVA framework, freely available at https://github.com/ovttiras/QSAR_KRAS_inhibitors_v2/blob/main/KRASAVA%20v2.ipynb (accessed on 10 December 2025), a reproducible Jupyter Notebook, executable via Google Colab. In KRASAVA, one can enter the information on the chemical structures of compounds under investigation via the SMILES linear notations [34], or files in CSV or SDF format. One of the processing steps in KRASAVA is the automatic validation and standardization of the input chemical structures. If a user enters an invalid structure, the application returns the index number of the compound in the uploaded CSV or SDF file, along with the SMILES notation of the corresponding structure.

For chemical structures that successfully pass validation and standardization, the application checks for the availability of experimental IC_50_ values for KRAS G12D in the ChEMBL database. If experimental data are available, the framework displays the mean value, standard deviation of the experimental IC_50_ values, and the corresponding ChEMBL compound ID [35]. In this case, activity prediction is not performed.

**Figure 3:**
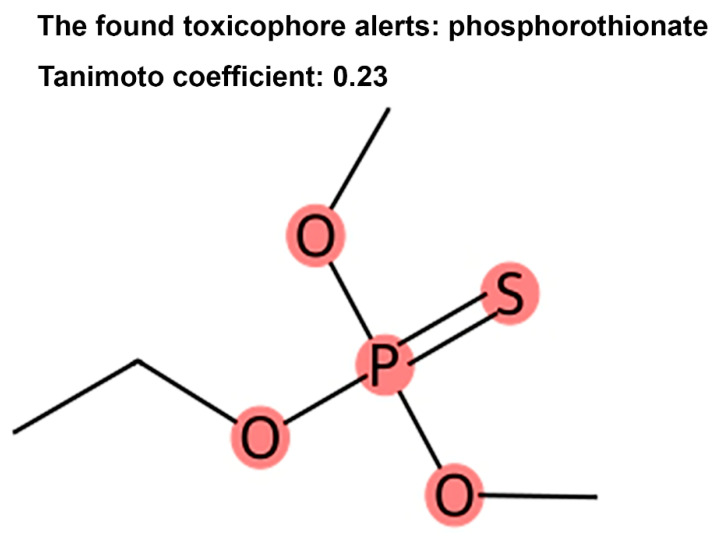
Highlighting of the identified structural fragment that consistently increases acute oral toxicity in rats. When a toxicophore is detected, the Tanimoto similarity index of the fragment to the molecule is also returned.

When analyzing individual compounds, the KRASAVA framework implements the assessment of compliance with Muegge’s rules. Previously, we proposed a set of structural fragments—toxicophores—that consistently increase the level of acute oral toxicity in rats [36]. We integrated the identification of these fragments, along with Brenk filters and PAINS, into the KRASAVA framework (Figure 3), allowing preliminary evaluation of oral bioavailability and potential toxicity.

In addition, the framework includes identification of molecular fragments associated with the most significant molecular descriptors (Figure 2) that were determined through structural interpretation for the purposes of molecular design.

Based on the identified structure-activity relationships and using the capabilities of the KRASAVA framework, we performed a rational molecular design using the compound 4-(3,8-diazabicyclo[3.2.1]octan-3-yl)-8-fluoro-2-[[(2R,8S)-2-fluoro-1,2,3,5,6,7-hexahydropyrrolizin-8-yl]methoxy]-7-[5-methoxy-2-(trifluoromethoxy)phenyl]pyrido [4,3-d]pyrimidine (PubChem CID 156124915, SCHEMBL23053462, BDBM573509, Example 361), having the pIC_50_ value of 5.57, according to the patent US-11453683-B1 [37].

**Figure 4:**
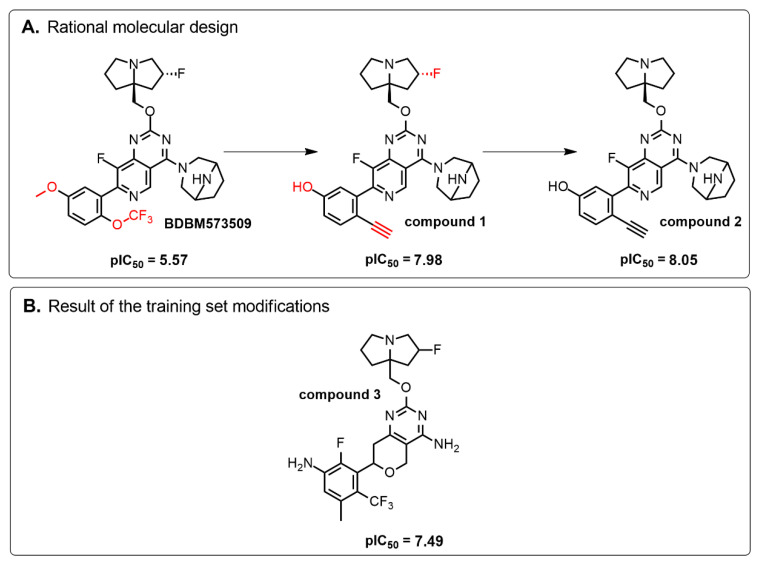
Promising inhibitors of KRAS G12D. (**A**) Rational molecular design based on the identified structure-activity relationships of KRAS G12D inhibitors (see Table 2, Figure 2). (**B**) Compound 3 was proposed as a result of the combinatorial modification of the functional groups of the compounds from the training set. Molecular transformations are highlighted in red in chemical structures.

If the structure of compound BDBM573509 is modified by replacing the methoxy group with a hydroxy group (pattern No. 4) and the trifluoromethoxy group (a methoxy derivative) with an alkyne group (pattern No. 2), the inhibitory activity increases—the calculated pIC_50_ value for the new compound (compound **1**) is 7.98 (Figure 4). In the modified compound **1**, unlike the original molecule BDBM573509, pattern No. 6 is also satisfied, according to which a hydroxy group in the para-position relative to other substituents enhances the inhibitory activity against KRAS G12D.

Further modification, specifically the elimination of the fluorine atom initially located as a substituent in the pyrrolizidine ring, also increases the inhibitory activity—for compound **2**, the predicted pIC_50_ value is 8.05 (Figure 4). According to the ChEMBL database, the experimental pIC_50_ value for the known KRAS G12D inhibitor MRTX1133 (PubChem CID 162369732, CHEMBL5081048, PDB ID 6IC) is 8.25 ± 0.47.

In addition, to optimize bioavailability, we performed modifications of the training set compounds and obtained the proposed compound **3** (Figure 4) with a predicted pIC_50_ value of 7.49. Using the KRASAVA framework, we predicted the inhibitory activities of compounds **1**–**3** by considering their inclusion within the applicability domain of the consensus QSAR model.

For comparative analysis of predictive performance, we also calculated the inhibitory activities of compounds **1**–**3** using the best QSAR model developed on the OCHEM platform (https://ochem.eu/model/20748154) (accessed on 10 December 2025). For compound **1** and compound **2**, this model predicts a pIC_50_ value of 8.80, while for compound **3**, the predicted value is 8.1.

**Figure 5:**
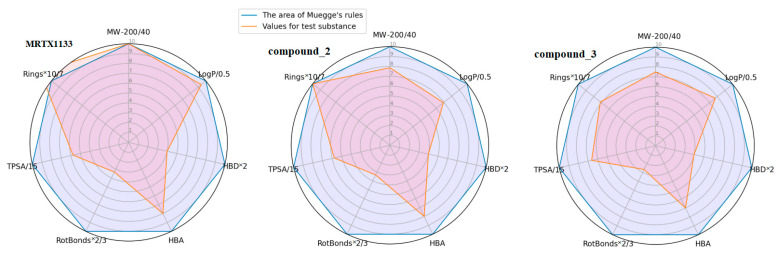
Visualization of the compliance of the investigated compounds with Muegge’s rules using bioavailability radar plots.

Compared to MRTX1133, the investigated compounds **2** and **3** (Figure 5 and Table 3) exhibit a better combination of physicochemical properties, suggesting a potentially acceptable level of bioavailability.

It should be noted that we did not find the structures and experimental IC_50_ values for the investigated compounds 1–3 in the SureChEMBL (https://www.surechembl.org/) (accessed on 10 December 2025), PubChem (https://pubchem.ncbi.nlm.nih.gov/) (accessed on 10 December 2025), or BindingDB (https://www.bindingdb.org/rwd/bind/index.jsp) (accessed on 10 December 2025) databases. However, further studies are required to assess the patent landscape for compounds **1**–**3** through a detailed analysis of Markush structures in existing patents. Moreover, compounds **1** and **3** are considered solely as examples of rational molecular design based on the structural interpretation of QSAR models, aimed at the balanced search for active KRAS G12D inhibitors with acceptable bioavailability.

**Figure 6:**
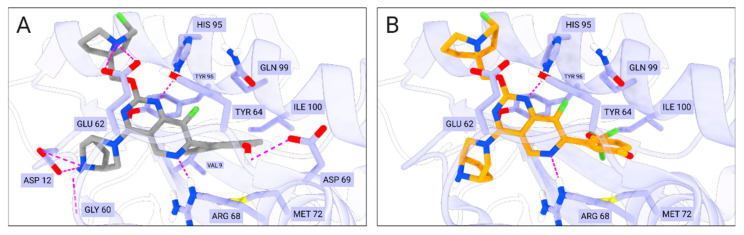
Key interactions of amino acid residues in the active site of the KRAS G12D 7T47 model (violet) with (**A**) co-crystalized ligand MRTX1133 (gray) and (**B**) BDBM573509 (yellow). Ionic and hydrogen bonds are shown as magenta dashed lines. Gly60 interacts with the ligand via the oxygen backbone atom. Additionally, the side chains of the amino acids of the active site that interact with a ligand via hydrophobic contacts are displayed.

For comparative analysis of the QSAR modeling results, we additionally performed molecular docking of compound BDBM573509, compounds **1**–**3**, using MRTX1133 as a reference compound. It binds to a specific pocket (S-IIP) on the surface of mutant KRAS G12D, which forms only in the presence of the mutation and in the inactive (GDP-bound) state of the protein. According to [38], MRTX1133 forms key interactions with Asp69, Asp12, Gly60, Glu62, His95, Arg68, as well as weaker interactions with Gln99, Glu63, and Ser65 (Figure 6).

Table 4 presents the results of molecular docking for compound BDBM573509, compounds **1**–**3**, and MRTX1133. According to the data in Table 4, there is a weak correlation between the pIC_50_ values and the docking scores. Previously, it was shown that molecular docking was explored in parallel with QSAR modeling, but molecular docking failed to correctly discriminate between experimentally active and inactive compounds [39]. In this regard, the priority task in this study was not to calculate the values of docking scores, but primarily to analyze the interactions of the studied compounds with key amino acid residues in the active center of the KRAS G12D enzyme.

**Figure 7:**
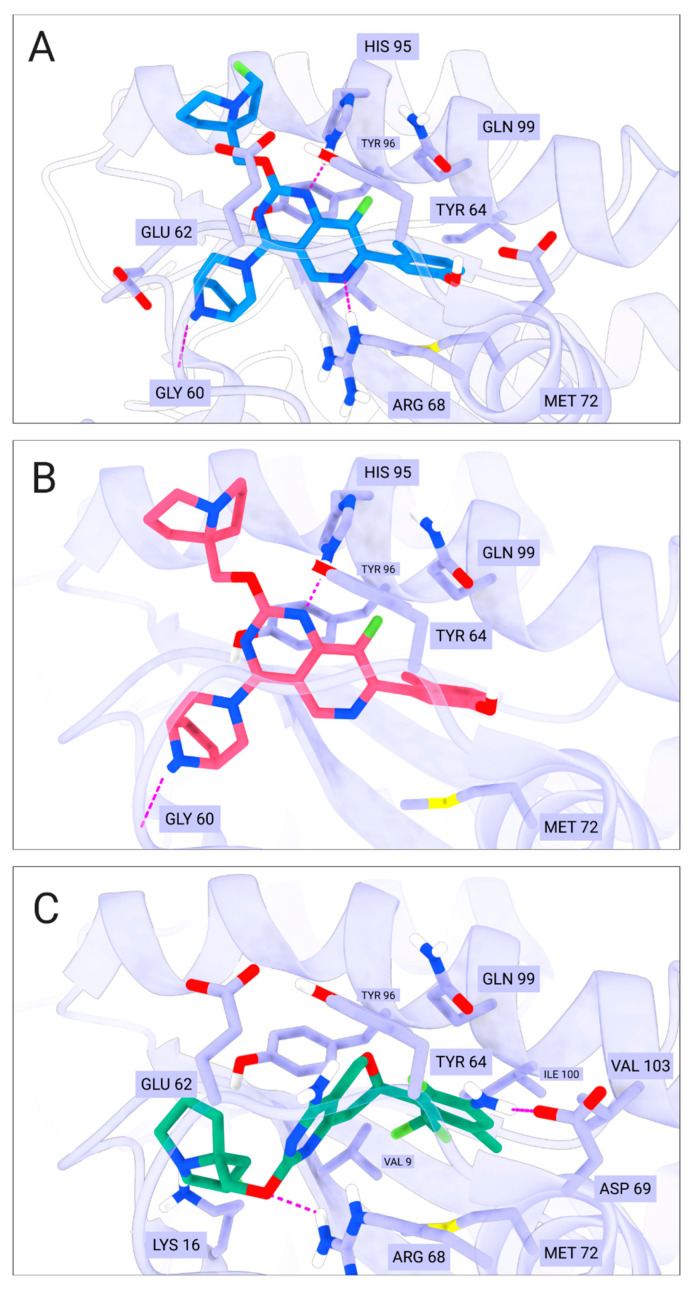
Key interactions of amino acid residues in the active site of the KRAS G12D 7T47 model (violet) with (**A**) compound 1 (blue), (**B**) compound **2** (pink), and (**C**) compound **3** (green). Ionic and hydrogen bonds are shown as magenta dashed lines. Gly60 interacts with the ligand via the oxygen backbone atom. Additionally, the side chains of the amino acids of the active site that interact with a ligand via hydrophobic contacts are displayed.

**Figure A1:**
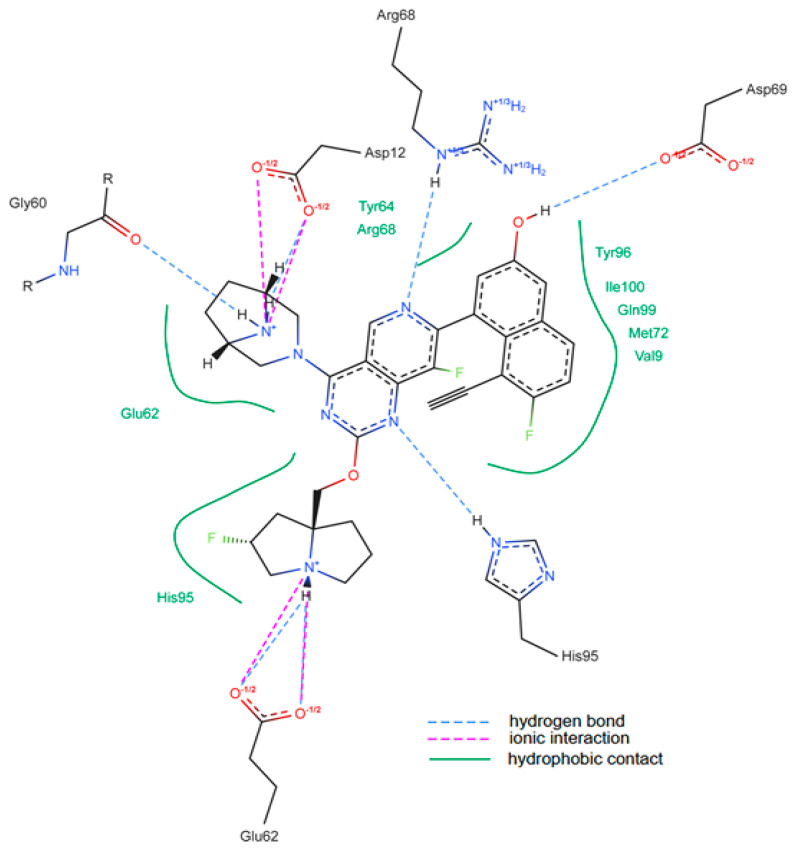
Key interactions of the co-crystallized ligand MRTX1133 with amino acid residues in the active site of the KRAS G12D 7T47 model.

**Figure A2:**
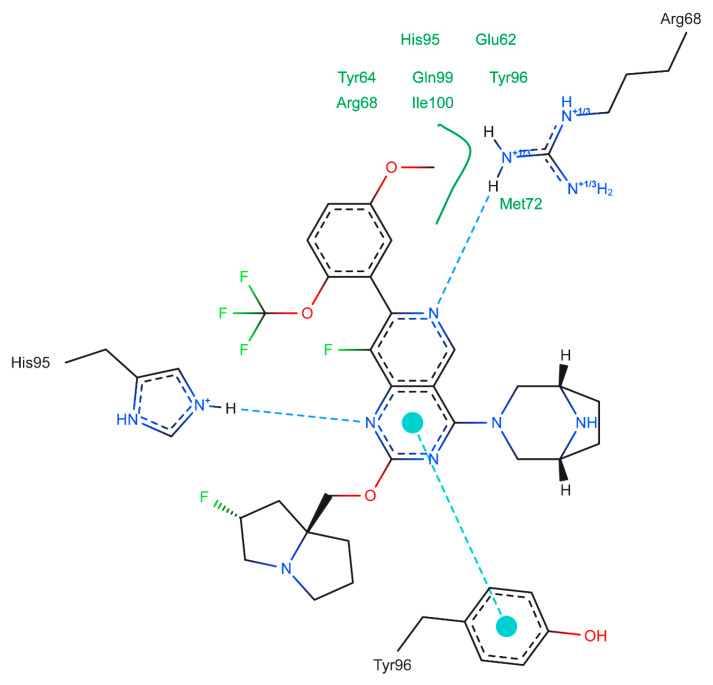
Key interactions of the co-crystallized ligand BDBM573509 with amino acid residues in the active site of the KRAS G12D 7T47 model.

**Figure A3:**
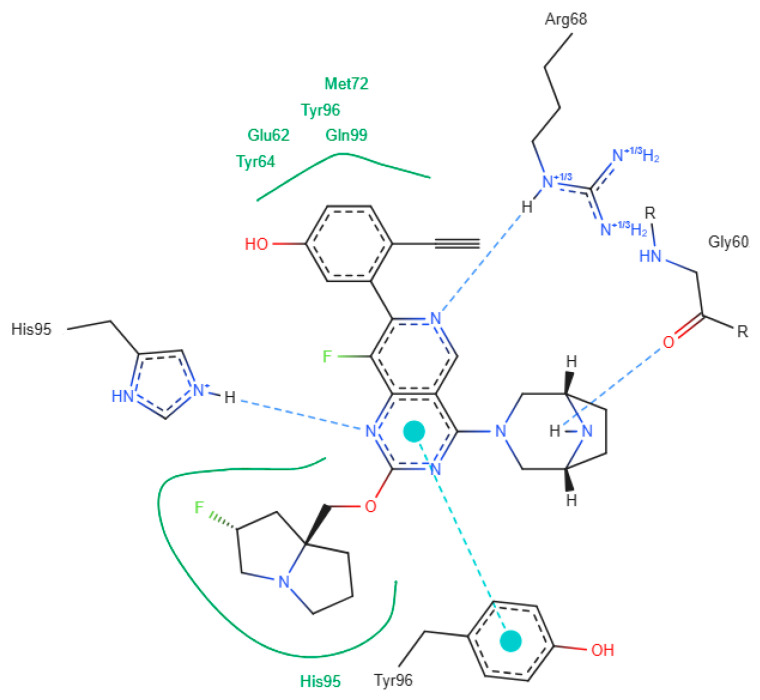
Key interactions of compound **1** with amino acid residues in the active site of the KRAS G12D 7T47 model.

**Figure A4:**
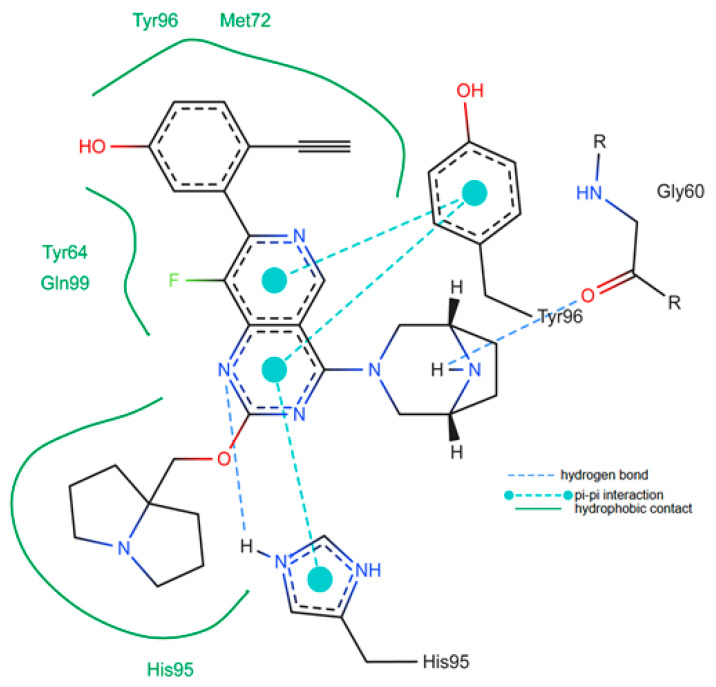
Key interactions of compound **2** with amino acid residues in the active site of the KRAS G12D 7T47 model.

**Figure A5:**
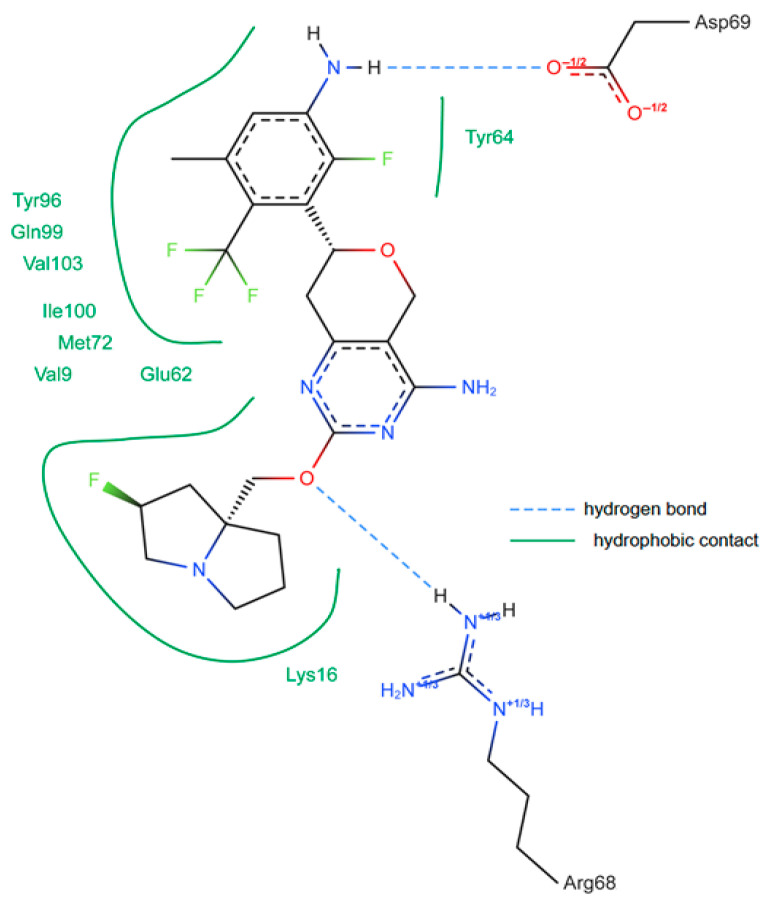
Key interactions of compound **3** with amino acid residues in the active site of the KRAS G12D 7T47 model.

Figure 6, Figure 7, Figure A1, Figure A2, Figure A3, Figure A4 and Figure A5 show the interactions of compound BDBM573509, compounds **1**–**3**, and MRTX1133 with key amino acid residues in the KRAS G12D active site. One can see the following key interactions from Figure A1, Figure A2, Figure A3, Figure A4 and Figure A5:

- Ionic interactions with Asp12—compound MRTX1133;
- Gly60 hydrogen bond—compound MRTX1133, compound **1** and compound **2**;
- His95 hydrogen bond—compounds MRTX1133, BDBM573509, compound **1** and compound **2**;
- Arg68 hydrogen bond—compounds MRTX1133, BDBM573509, compound **1** and compound **3**;
- Asp69 hydrogen bond—compound MRTX1133 and compound **3**.

The 2D visualization of the interactions of compound BDBM573509, compounds **1**–**3**, and MRTX1133 with key amino acid residues is provided in Appendix A (Figure A1, Figure A2, Figure A3, Figure A4 and Figure A5). The identified interactions are supported by previous studies on similar compounds [38].

Analyzing the data in Table 2 and Figure 2 and Figure 6, we noted a consistent correlation between the structural interpretation of the QSAR models and molecular docking results. According to the QSAR model interpretation, descriptors describing the hydroxy substituent (descriptors No. 2 and 4 in Figure 2A, as well as descriptor No. 5 in Figure 2B) contribute significantly to activity, which is confirmed by the formation of hydrogen bonds with Asp69 in MRTX1133.

The present study of inhibitors has several distinctive features compared to existing studies also devoted to QSAR analysis of KRAS inhibitors. The main advantage of the present study compared with previous works by Srisongkram et al. [28,29] is twofold. First, we have developed and validated QSAR models for KRAS G12D inhibitors, which are of greater medicinal chemistry interest than the KRAS G12C inhibitors investigated by Srisongkram et al. The development of KRAS G12D inhibitors is particularly promising for the treatment of pancreatic and colorectal cancers, where the clinical need is high. Second, compared with the work of Srisongkram et al. [28,29], we propose a freely available framework that significantly automates the virtual screening of KRAS G12D inhibitors. A comparative analysis of this study with existing ones is presented in Table 5, which demonstrates that our research has several advantages. For example, in this study, when developing QSAR regression models, we defined the applicability range, performed a structural interpretation, and, most importantly, integrated the proposed consensus models into the KRASAVA framework, which enables intensified virtual screening of KRAS G12D inhibitors, taking into account preliminary assessments of oral bioavailability and toxicity. The developed QSAR models (Table 1), as well as the algorithms for their construction, statistical characteristics in the form of Jupyter Notebook program files, and the results of molecular docking, are freely available at the GitHub repository https://github.com/ovttiras/QSAR_KRAS_inhibitors_v2 (accessed on 10 December 2025).

## 3. Methods and Materials

### 3.1. Dataset of KRAS G12D Inhibitors

For QSAR modeling, we extracted a dataset of compounds with experimentally reported IC_50_ values against KRAS G12D from publication [40]. In accordance with established recommendations for data curation in cheminformatics [33], we verified and preprocessed the initial dataset, consisting of 645 compounds and publicly available at https://zenodo.org/records/11137638 (accessed on 10 December 2025). Mixtures, compounds with incorrect or inconsistent chemical structures, and salts were excluded from further consideration. During the curation process, nine structurally invalid entries and four salt forms were identified and removed. The original Python code used for dataset validation is publicly available at https://github.com/ovttiras/QSAR_KRAS_inhibitors_v2/blob/main/DATA%20curation%20and%20cleaning.ipynb (accessed on 10 December 2025).

Since the objective of this study was to construct regression QSAR models capable of predicting IC_50_ values toward KRAS G12D, only entries containing explicit numerical IC_50_ values were retained; records with non-numeric values containing “>” or “<“ qualifiers were excluded. Experimental bioactivity values expressed in molar IC_50_ were converted into their negative decimal logarithm (pIC_50_), which is conventionally used in QSAR studies due to its improved linearity with respect to biological response.

For compounds having two or more reported experimental pIC_50_ measurements, we calculated the mean and standard deviation. We retained only entries with a standard deviation not exceeding 0.5 log units, in accordance with a previously described procedure for handling duplicate measurements [41].

To evaluate the predictive performance of the QSAR models, we divided the initial dataset comprising 566 compounds into training (ws) and test (ts) sets. The final sorted dataset was ordered by increasing pIC_50_, and every fifth compound was assigned to the test set, while the remaining entries formed the training set. As a result, the training set and the test set comprised 452 and 114 compounds, respectively. We exported both subsets into SDF format using RDKit v 2025.03.6 in Python v3.11; they are publicly available at https://github.com/ovttiras/QSAR_KRAS_inhibitors_v2/tree/main/datasets (accessed on 10 December 2025).

**Figure 8:**
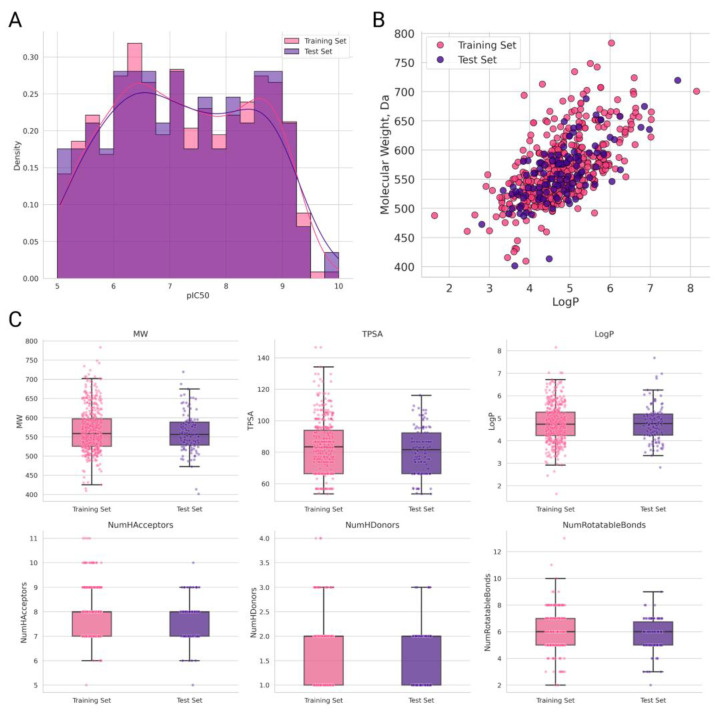
Description of the training and the test sets. (**A**) Distribution of experimental pIC_50_ values for the training and the test sets. (**B**) Visualization of the distribution of chemical space in the molecular weight (MW)—lipophilicity (LogP) coordinate system for the training and the test sets. (**C**) Visualization of the chemical space.

The distribution of activity values in the training and test sets is shown in Figure 8A. As can be seen, the two sets exhibit similar ranges and frequencies of experimental pIC_50_ values. The investigated compounds cover a sufficiently broad pIC_50_ range of more than five logarithmic units, which has a positive effect on the descriptive and predictive performance of the developed QSAR models. As previously demonstrated in [42], an adequate QSAR model requires an activity range of at least one logarithmic unit.

Visualization of the chemical space of the training and test sets in the molecular weight (MW)—lipophilicity (LogP) coordinate system is presented in Figure 8B. Analysis of this plot indicates a sufficiently high level of chemical diversity in both subsets, as evidenced by the wide ranges of molecular weight and lipophilicity of the investigated compounds.

In order to further assess the predictive power of the developed models, a second external test set was formed by aggregating compounds with experimental pIC_50_ values for KRAS G12D from the ChEMBL [35], BindingDB [43] databases, and a study [44] devoted to the development of KRAS G12D inhibitors. When collecting data, the data verification and preprocessing methodology described above was applied. The total volume of the second independent test sample was 1266 compounds.

### 3.2. Development of QSAR Models

Molecular structures were described using ECFP4 (radius = 2, nBits = 1024, useFeatures = False, useChirality = False), MACCS (166-bit structural keys), Klekota-Roth, PubChem descriptors, Topological Torsion, Atom Pairs, and Topological Path-Based fingerprints. Descriptor calculation was performed using the RDKit v2025.03.6 [45] and PaDEL-Descriptor v0.1.11 (PadelPy) [46,47] libraries in Python v3.11. We built QSAR models using the scikit-learn [48] and CatBoost v1.2.6 [49] libraries, applying gradient boosting (CatBoost), support vector machines (SVM), and fully connected neural networks with a multilayer perceptron (MLP) architecture. Hyperparameters of the models were automatically optimized using GridSearchCV implemented in scikit-learn. Both the model hyperparameters and the implementation scripts in Jupyter Notebook format are publicly available at https://github.com/ovttiras/QSAR_KRAS_inhibitors_v2 and can be utilized for QSAR modeling of other types of biological activity.

To assess the robustness of the models, five-fold internal cross-validation (5-fold CV) was performed [50]. The inclusion of test set compounds within the applicability domain (AD) was evaluated using the similarity distance approach [51]. A test compound is considered to belong to the QSAR model’s applicability domain if its similarity distance does not exceed the threshold value D_c_, calculated using Equation (1):

(1) $$D_{c}=Z\sigma +\underline{y},$$

where $\underline{y}$ and σ are the mean and standard deviation of the Euclidean distances in the descriptor chemical space between all compounds in the training set and their nearest neighbors; Z is a constant, typically set to 0.5.

Data coverage (Cov) within the applicability domain was calculated as the ratio of the number of test set compounds falling within the AD to the total number of compounds in the test set. The predictive performance of the QSAR models was evaluated using the coefficient of determination (Q^2^):

(2) $$Q^{2}=1\;-\frac{\sum_{i}{\left(y_{i}-{\hat{y}}_{i}\right)}^{2}}{\sum_{i}{\left(y_{i}-y_{mean}\right)}^{2}},$$

And the root mean square error (RMSE):

(3) $$\mathrm{RMSE}=\sqrt{\frac{\sum_{i=1}^{m}{\left(y_{i}-{\hat{y}}_{i}\right)}^{2}}{m-1}},$$

where $y_{i}$ is the observed activity of the *i*-th compound, ${\hat{y}}_{i}$ is the predicted activity of the *i*-th compound, $y_{mean}$ is the mean observed activity, and *m* is the number of compounds in the dataset. We calculated the Q^2^ and RMSE values using the scikit-learn library.

To improve predictive power, we developed a consensus model. The predicted consensus activity value was calculated as the average of the predictions from the three best QSAR models.

For validation of adequate QSAR models, the y-randomization method with 50 iterations was applied using the permutation_test_score module in the scikit-learn library. The absence of random correlation in the QSAR models was estimated using the Q2_rand determination coefficient and *p*-value. If a *p*-value is sufficiently small, usually below a certain threshold (e.g., 0.05), there is no random correlation in the relationship described by a model [52].

During structural interpretation, we evaluated the contribution of descriptors to the QSAR models using the SHAP library in Python v3.11 [53]. We assessed feature importance in SHAP v0.44.0 based on Shapley values, which quantify the contribution of each descriptor to the model predictions.

For a comparative analysis of the predictive performance of the developed QSAR models of KRAS inhibitors, we additionally employed the OCHEM web platform (https://ochem.eu) (accessed on 10 December 2025), which provides various sets of molecular descriptors and machine learning algorithms. The OCHEM platform integrates several software packages for calculating a wide range of descriptor sets. In this study, we used eleven descriptor sets for QSAR model development, including OEState [54] combined with AlogPS [55], CDK [56], Dragon v7 [57], QNPR descriptors [58], Extended Connectivity Fingerprint 4 (ECFP4) [59], alvaDesc [60], Fragmentor (length: 2–4) [61], MOLD2 [62], and MORDRED [63]. QSAR models were developed using various machine learning methods, including Associative Neural Networks (ASNN) [55], Random Forest, Gradient Boosting implemented in the XGBoost library [64], Deep Neural Networks (DNN) [65], k-Nearest Neighbors (KNN) [66], and Multiple Linear Regression Analysis (MLRA) [67]. In addition, several deep learning approaches were applied: Transformer Convolutional Neural Network (TRANSNNI) [68], Attentive FP [69], and Chemprop [70]. The models were built using optimized parameter settings for each machine learning method provided by the OCHEM platform. The applicability domain was assessed using the “distance-to-model” concept, specifically the “BAGGING-STD” approach described in the OCHEM user manual [71].

### 3.3. Framework KRASAVA

We implemented the consensus QSAR model in KRASAVA (https://github.com/ovttiras/QSAR_KRAS_inhibitors_v2/blob/main/KRASAVA%20v2.ipynb) (accessed on 10 December 2025), a Python-based framework realized as a reproducible Jupyter Notebook, executable via Google Colab. The KRASAVA framework was created in Python v3.11 and utilizes the RDKit v2025.03.6 [45], scikit-learn v1.3.1 [48], NumPy v1.22.1 [72], and Pandas v1.3.5 [73] libraries.

### 3.4. Molecular Docking

We used GNINA v1.3 [74] because its machine-learning-based scoring functions provide superior performance compared to Vina. Although Gnina follows the same docking workflow as Vina, it achieves higher accuracy by rescoring ligand poses with convolutional neural network scoring functions after the initial Vina scoring. This additional ML-based rescoring step improves the reliability of binding pose predictions. Gnina demonstrated strong performance in practical applications [75] and independent benchmark studies [76], further supporting its reliability.

To validate the selected docking protocol, a re-docking procedure was conducted, in which the best docking pose of the ligand was superimposed onto the co-crystallized ligand conformer, and the root-mean-square deviation (RMSD) was measured. According to [77], the generally accepted RMSD threshold for re-docking, given the chosen preprocessing and docking protocol, should be ≤2 Å.

For molecular docking of potential KRAS G12D inhibitors, the 7T47 protein model (resolution: 1.27 Å, single chain A) was selected from the RCSB Protein Data Bank (https://www.rcsb.org/) (accessed on 10 December 2025), as it contains a co-crystallized KRAS G12D inhibitor MRTX1133 (PubChem CID: 162369732, CHEMBL5081048, PDB Chemical Component ID: 6IC), which served as a reference compound [11].

Ligand and protein preprocessing were performed in Chimera [78] using the Dock Prep module. Ligand preprocessing included protonation and geometry optimization using the GAFF2/AM1-BCC force field. During the preprocessing of the molecular target, the native 7T47 protein model was cleaned of co-crystallized ligands (GCP, GDP, glycerol, and acetate ion), water molecules, and the magnesium ion. Chain A was protonated at pH 7.4, partial charges were added, and missing amino acid residues were restored.

In GNINA, the binding site was defined based on the position of MRTX1133 (−23.00 Å; 5.14 Å; 23.02 Å) using the autobox_ligand option with default parameters (exhaustiveness = 16, num_modes = 9) and a 4 Å margin in all directions.

**Figure 9:**
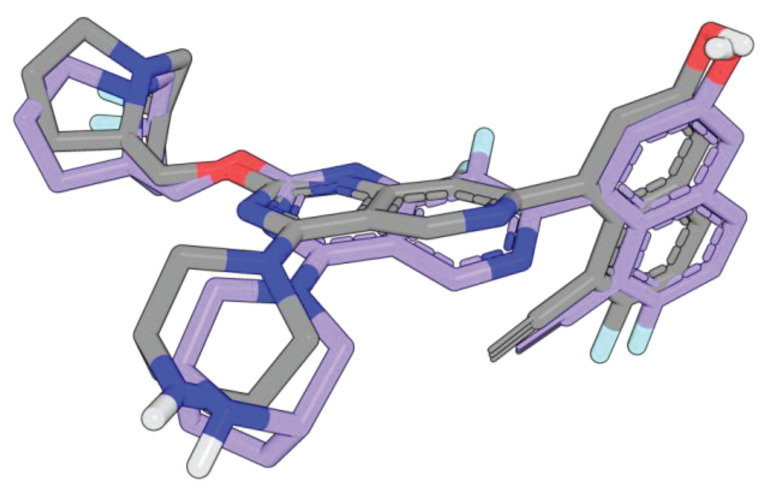
Superimposition of the docked ligand (violet) and the co-crystallized ligand MRTX1133 (gray) during validation of the docking procedure for the protein model 7T47 PDB. The obtained RMSD value was 0.76 Å.

During the re-docking of MRTX1133, the RMSD value was 0.76 Å (Figure 9), indicating that the applied docking protocol successfully reproduces ligand conformations consistent with crystallographic data. The RMSD value was calculated using the mcs_rmsd function implemented in the useful_rdkit_utils library v 0.93 [79].

Molecular docking results were visualized using the ProteinsPlus web application [80]. All docking data are publicly available at https://github.com/ovttiras/QSAR_KRAS_inhibitors_v2/tree/main/Docking (accessed on 10 December 2025).

## 4. Conclusions

The conducted study enables the following outcomes:

- Development of a series of regression QSAR models for KRAS G12D inhibitors using ECFP4, Klekota-Roth, PubChem, MACCS, Topological Torsion, Atom Pairs, Topological Path-Based fingerprints, and RDKit descriptors, along with CatBoost, SVM, and MLP algorithms, as well as models developed via the OCHEM platform;
- Structural interpretation of QSAR models for KRAS G12D inhibitors, identifying the most significant fragments and molecular transformations;
- Integration of the consensus QSAR model into the KRASAVA framework, enabling retrieval of experimental data for investigated compounds, as well as virtual screening of potential KRAS G12D inhibitors with preliminary assessment of bioavailability through Muegge’s rules compliance, and evaluation of acute toxicity via identification of key toxicophores and Brenk filters;
- Rational molecular design of compounds based on the structural interpretation results and capabilities of the KRASAVA framework, leading to the proposal of two most promising KRAS G12D inhibitors;
- Comparative analysis of the proposed compounds through molecular docking, examining the nature of their interactions with the KRAS G12D binding site, and validating the results obtained from QSAR structural interpretation.

The results of this study are expected to reduce financial, temporal, and labor costs associated with the synthesis and testing of new KRAS G12D inhibitor drugs. We consider experimental validation an important direction for future studies.

## Figures and Tables

**Table 1:** Statistical characteristics of the developed QSAR models of KRAS inhibitors.

Descriptors	Algorithms	Training Set, 5-Fold CV		Test Set				
				All Compounds		Cov	AD Compounds	
		$Q_{cv}^{2}$	RMSE	$Q_{ts}^{2}$	RMSE		$Q_{ts}^{2}$	RMSE
Topological Torsion fingerprints	CatBoost	0.65	0.73	0.67	0.71	0.75	0.61	0.74
	SVM	0.66	0.71	0.65	0.73		0.60	0.74
	MLP	0.63	0.74	0.60	0.79		0.55	0.79
MACCS	CatBoost	0.48	0.88	0.46	0.91	0.67	0.37	0.97
	SVM	0.48	0.88	0.46	0.91		0.37	0.98
	MLP	0.44	0.91	0.44	0.93		0.36	0.99
PubChem	CatBoost	0.58	0.79	0.65	0.73	0.74	0.59	0.75
	SVM	0.60	0.78	0.60	0.78		0.49	0.85
	MLP	0.57	0.80	0.60	0.78		0.51	0.81
KlekotaRoth	CatBoost	0.62	0.76	0.66	0.72	0.81	0.65	0.72
	SVM	0.63	0.74	0.64	0.74		0.64	0.74
	MLP	0.50	0.86	0.64	0.75		0.63	0.75
Atom Pairs fingerprints	CatBoost	0.57	0.80	0.53	0.85	0.79	0.52	0.87
	SVM	0.61	0.76	0.56	0.82		0.61	0.79
	MLP	0.57	0.80	0.59	0.79		0.58	0.82
**ECFP4**	**CatBoost**	**0.65**	**0.73**	**0.69**	**0.68**	**0.78**	**0.66**	**0.70**
	SVM	0.68	0.69	0.68	0.70		0.66	0.70
	MLP	0.66	0.72	0.63	0.75		0.61	0.74
**Topological Path-Based fingerprints**	**CatBoost**	**0.60**	**0.77**	**0.64**	**0.75**	**0.82**	**0.67**	**0.70**
	SVM	0.65	0.72	0.62	0.76		0.62	0.76
	MLP	0.54	0.82	0.58	0.80		0.56	0.81
**RDKit**	**CatBoost**	**0.64**	**0.73**	**0.65**	**0.73**	**0.82**	**0.67**	**0.71**
	SVM	0.60	0.77	0.58	0.81		0.62	0.76
	MLP	0.51	0.86	0.52	0.86		0.53	0.85
**Consensus** (ECFP4 + Topological Path-Based fingerprints + RDKit)	CatBoost	0.68	0.69	0.71	0.69	0.78	0.70	0.66

**Table 2:** Top 10 molecular transformations (MT) affecting KRAS inhibitory activity.

#	Molecular Transformations and SMIRKS	# MT	ΔMean	An Example of a Molecular Transformation (Molecular Pair)
Reducing Inhibitory Activity				
1	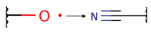 [O] * -> * C#N	8	−2.0 ± 0.85	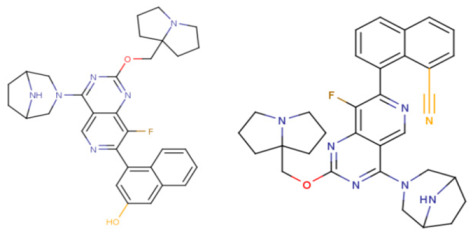 pIC_50_ = 9.30 pIC_50_ = 7.69
2	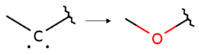 [C][C] * -> [C]O *	5	−1.9 ± 1.0	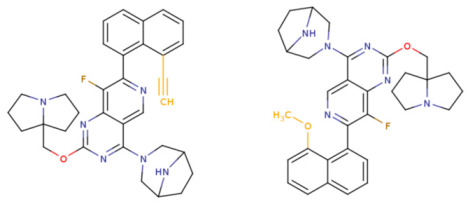 pIC_50_ = 8.96 pIC_50_ = 7.60
3	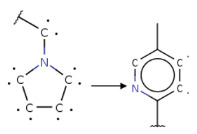 [C]N1[C][C][C][C@H]1 * -> [C]c1[c][c]c(*)n[c]1	5	−1.5 ± 0.23	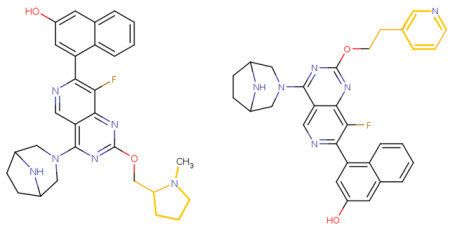 pIC_50_ = 8.57 pIC_50_ = 6.81
4	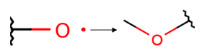 [O] * -> [C]O *	9	−1.4 ± 1.5	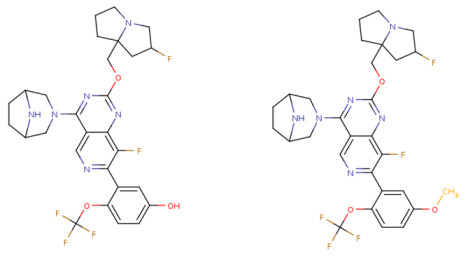 pIC_50_ = 9.40 pIC_50_ = 5.57
5	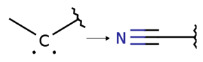 [C][C] * -> * C#N	4	−1.4 ± 0.63	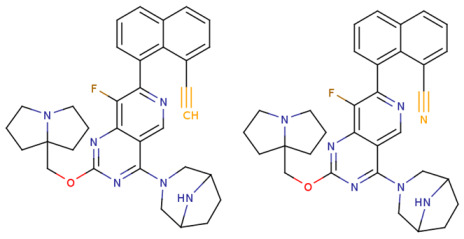 pIC_50_ = 8.96 pIC_50_ = 7.69
**Increasing inhibitory activity**				
6	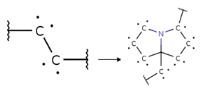 * [C][C] * -> * [C]C12[C][C][C]N1[C](*)[C][C]2	6	25 ± 0.22	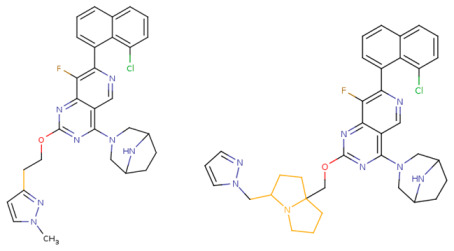 pIC_50_ = 5.30 pIC_50_ = 8.12
7	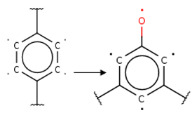 * c1[c][c]c(*)[c][c]1 -> [O]c1[c]c(*)[c]c(*)[c]1	4	2.0 ± 0.2	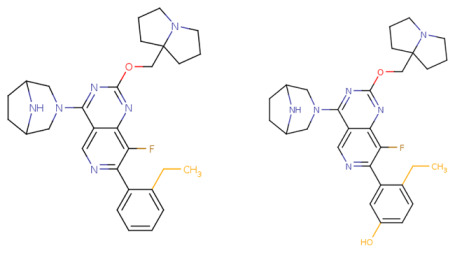 pIC_50_ = 6.71 pIC_50_ = 8.82
8	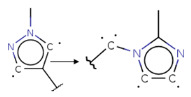 * c1[c][c]n[c][c]1 -> [C]c1n[c][c]n1[C] *	4	1.8 ± 0.36	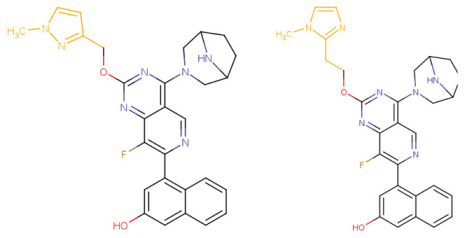 pIC_50_ = 5.82 pIC_50_ = 8.02
9	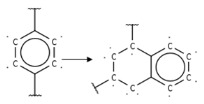 * c1[c][c]c(*)[c][c]1 -> * [C]1[C][C](*)c2[c][c][c][c]c2[C]1	11	1.8 ± 1.0	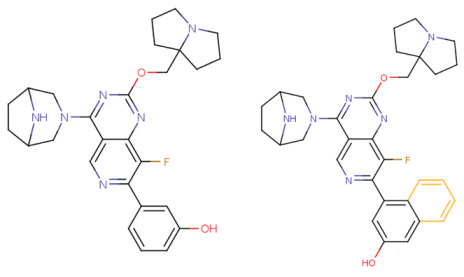 pIC_50_ = 5.79 pIC_50_ = 9.30
10	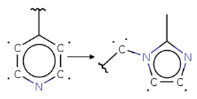 * c1[c][c]n[c][c]1 -> [C]c1n[c][c]n1[C] *	4	1.6 ± 0.3	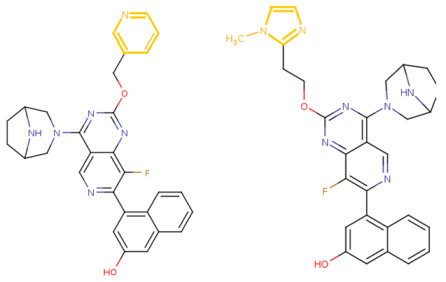 pIC_50_ = 6.18 pIC_50_ = 8.02

# is number; the yellow color in chemical structures highlights molecular transformations.

**Table 3:** Key physicochemical parameters of the investigated compounds and reference values according to Muegge’s bioavailability rules.

Parameters	MRTX1133	Compound 2	Compound 3	Muegge Rules
Molecular weight(MW), Da	600.7	514.6	499.5	200–600
Octanol-water coefficient(LogP)	4.71	3.47	3.88	≤5
Number of hydrogen bond donors (HBD)	2	2	2	≤5
Number of hydrogen bond acceptors(HBAs)	8	8	7	≤10
Number of rotatable bonds	5	5	4	≤15
Topological polar surface area (TPSA), Å^2^	86.64	86.64	99.52	≤150
Number of rings	8	7	5	≤7

**Table 4:** Molecular docking results.

Compound	pIC_50,_ * Experimental Data	Affinity, kcal/mol	Intra, kcal/mol	CNN Pose Score	CNN Affinity, pK
BDBM573509	5.57 *	−10.51	−0.34	0.6809	8.134
compound **1**	7.98	−11.81	−0.91	0.7935	8.193
compound **2**	8.05	−11.70	−0.87	0.7585	8.094
compound **3**	7.49	−8.44	3.64	0.6612	7.348
MRTX1133	8.25 ± 0.47 *	−13.55	−0.55	0.8120	8.554

**Table 5:** Comparative analysis of QSAR studies on KRAS G12D inhibitors.

Parameter for Comparison	Panik et al. [30]	Ajmal et al. [31]	This Study
Type of developed QSAR models	Binary classification	Binary classification	Regression
Description of the experimental data preprocessing procedure in accordance with mandatory requirements [33]	No	No	Yes
Molecular descriptors	PubChem	2D MOE	ECFP4, Klekota-Roth, PubChem, MACCS, Topological Torsion, Atom Pairs, Topological Path-Based fingerprints, RDKit, OEState, ALogPS, CDK, Dragon, QNPR, alvaDesc, Fragmentor, MOLD2, MORDRED
Machine learning methods	Random forest, k-nearest neighbors, support vector machine, XGBoost, LightGBM, CatBoost	Random forest, k-nearest neighbors, support vector machine	Random forest, k-nearest neighbors, support vector machine, XGBoost, LightGBM, CatBoost, Multilayered perceptron, deep neural network, associative neural networks, multiple linear regression analysis, transformer convolutional neural network, Attentive FP, Chemprop
Definition of the applicability domain —the third mandatory principle of QSAR modeling according to OECD [32]	No	No	Yes
Structural interpretation—the fifth recommended principle of QSAR modeling according to OECD [32]	No	No	Yes
Application of y-randomization for the identification of chance correlation	No	No	Yes
Form of QSAR model implementation	No	No	Jupyter Notebook, executable via Google Colab

## Data Availability

The data presented in this study are openly available in the [GitHub repository] at [https://github.com/ovttiras/QSAR_KRAS_inhibitors_v2] (accessed on 10 December 2025), reference number [ovttiras/QSAR_KRAS_inhibitors_v2].

## Abbreviations

The following abbreviations are used in this manuscript:

QSAR	Quantitative Structure–Activity Relationships
AD	Applicability Domain
5-fold CV	Five-fold Internal Cross-validation

## Appendix A

### Appendix A.1

**Table A1:** Statistical characteristics of the developed QSAR models of KRAS inhibitors.

Descriptors	Algorithms	Training Set, 5-Fold CV		Test Set, All Compounds	
		$Q_{cv}^{2}$	RMSE	$Q_{ts}^{2}$	RMSE
ALogPS, OEstate	RFR	0.57	0.80	0.59	0.79
	ASNN	0.59	0.78	0.50	0.87
	KNN	0.51	0.85	0.46	0.91
	MLRA	0	1.4	0.44	0.93
	DNN	0.58	0.79	0.50	0.91
	XGBOOST	0.57	0.80	0.59	0.79
CDK	RFR	0.61	0.76	0.57	0.81
	ASNN	0.59	0.78	0.53	0.85
	KNN	0.50	0.86	0.49	0.88
	MLRA	0.47	0.89	0	1.3
	DNN	0.58	0.80	0.50	0.89
	XGBOOST	0.61	0.76	0.60	0.79
Dragon	RFR	0.58	0.79	0.58	0.80
	ASNN	0.64	0.73	0.66	0.73
	KNN	0.51	0.85	0.53	0.85
	MLRA	0	1.5	0.36	0.99
	DNN	0.54	0.83	0.58	0.80
	XGBOOST	0.57	0.80	0.54	0.84
Fragmentor (length: 2–4)	RFR	0.60	0.77	0.62	0.77
	ASNN	0.55	0.82	0.61	0.77
	KNN	0.49	0.87	0.50	0.88
	MLRA	0.54	0.83	0.54	0.83
	DNN	0.45	0.91	0.5	0.85
	XGBOOST	0.57	0.80	0.60	0.79
MOLD2	RFR	0.59	0.78	0.58	0.80
	ASNN	0.59	0.78	0.50	0.84
	KNN	0.48	0.88	0.49	0.88
	MLRA	0.49	0.87	0	7
	DNN	0.45	0.91	0.52	0.86
	XGBOOST	0.58	0.79	0.59	0.79
MORDRED	RFR	0.59	0.78	0.58	0.8
	ASNN	0.61	0.76	0.67	0.71
	KNN	0.52	0.85	0.49	0.89
	MLRA	0	2.8	0	8
	DNN	0.51	0.85	0.5	0.88
	XGBOOST	0.57	0.80	0.57	0.81
QNPR	RFR	0.59	0.78	0.62	0.77
	ASNN	0.45	0.90	0.52	0.86
	KNN	0.38	0.96	0.36	0.99
	MLRA	0.42	0.93	0.52	0.86
	DNN	0.43	0.92	0.61	0.77
	XGBOOST	0.55	0.82	0.57	0.81
ECFP4	RFR	0.63	0.75	0.69	0.69
	ASNN	0.63	0.74	0.66	0.72
	KNN	0.56	0.81	0.60	0.79
	MLRA	0.23	1.07	0.4	1
	DNN	0.51	0.86	0.4	0.94
	XGBOOST	0.61	0.76	0.67	0.71
RDKIT	RFR	0.55	0.82	0.56	0.82
	ASNN	0.68	0.7	0.67	0.71
	KNN	0.53	0.84	0.51	0.87
	MLRA	0	2.4	0.3	1
	DNN	0.51	0.86	0.4	0.94
	XGBOOST	0.54	0.83	0.56	0.82
alvaDesc	RFR	0.59	0.78	0.59	0.79
	ASNN	0.65	0.72	0.65	0.73
	KNN	0.51	0.86	0.51	0.87
	MLRA	0	1.6	0	9
	DNN	0.53	0.83	0.56	0.82
	XGBOOST	0.59	0.78	0.63	0.76
-	AttFP AttFP	0.54	0.83	0.65	0.73
-	ChemProp	0.53	0.84	0.61	0.77
-	TRANSNNI	0.60	0.77	0.66	0.72

**Table A2:** Most significant Klekota-Roth descriptors for constructing QSAR models.

ID and SMARTS or Identifier of Substructure	Descriptor Visualization
KRFP1932 [!#1]c1[cH]c([!#1])c([!#1])c([!#1])[cH]1	
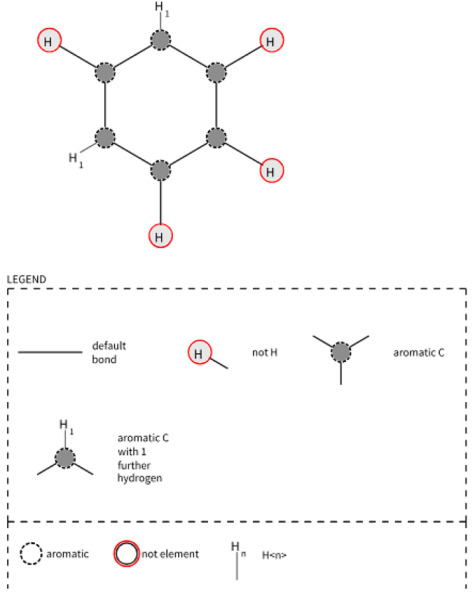
KRFP4740 Oc1ccc2ccccc2c1	
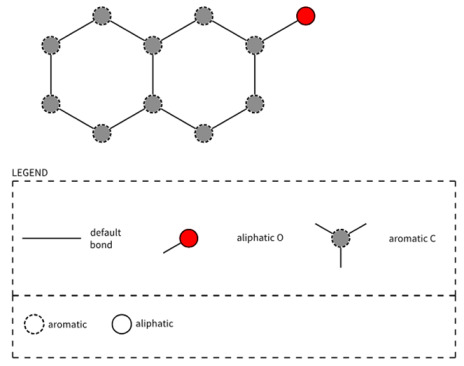
KRFP3139 c1ccc2ccccc2c1	
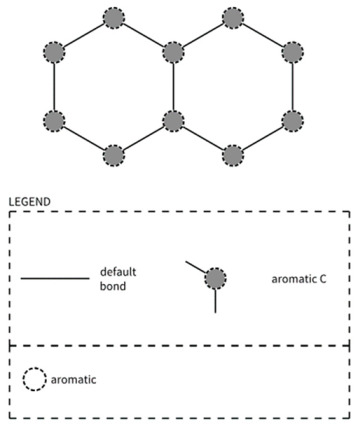
KRFP2949 [OH]	
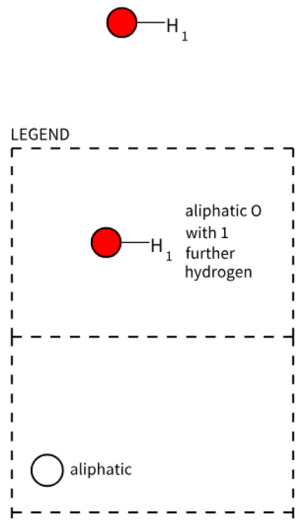
KRFP3592 Cc1cccc2ccccc12	
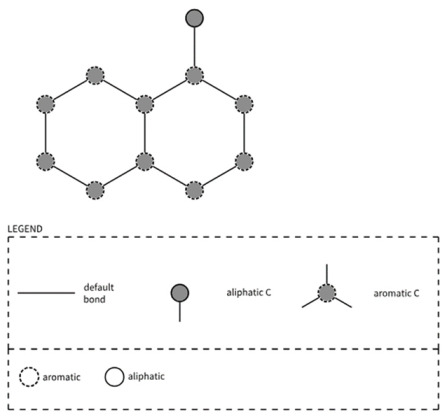
KRFP1566 [!#1]c1[cH][cH][cH][cH]c1[!#1]	
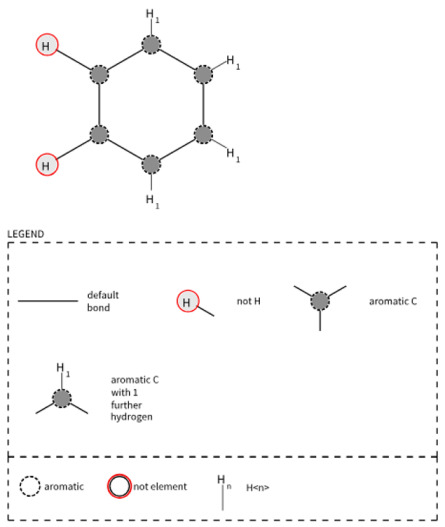
KRFP3751 CCN(C)C	
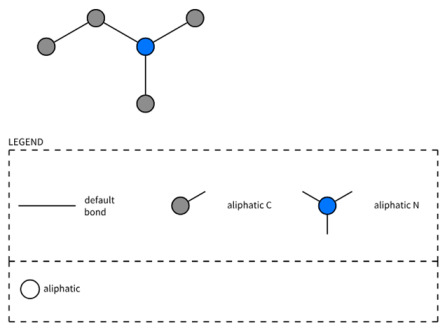
KRFP3719 CCCCCCC	
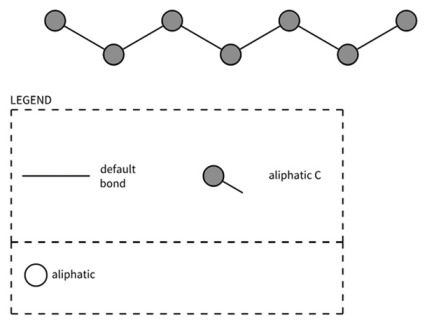
KRFP1148 [!#1][OH]	
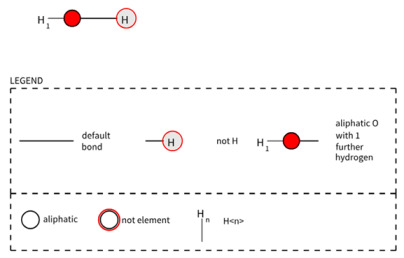

**Table A3:** Most significant PubChem fingerprints for constructing QSAR models.

ID	SMARTS or Identifier of Substructure	Descriptor Visualization
PubchemFP336	C(~C)(~C)(~C)(~N)	
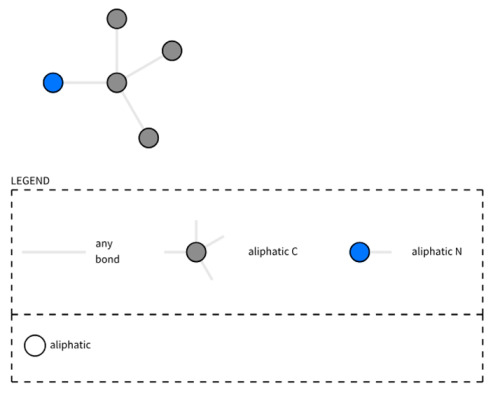
PubchemFP157	>=3 any ring size 5	
PubchemFP160	>=3 saturated or aromatic heteroatom-containing ring size 5	
PubchemFP590	C-C:C-O-[#1]	
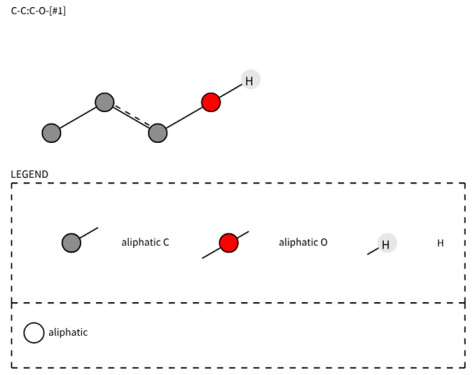
PubchemFP714	Cc1ccc(O)cc1	
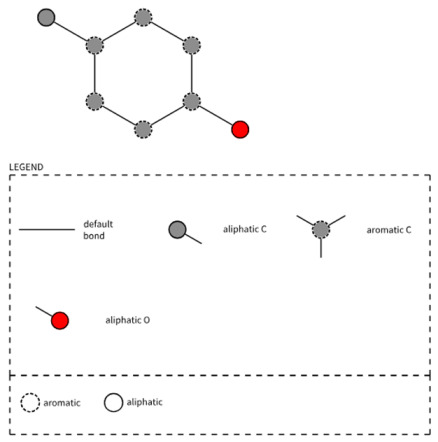
PubchemFP659	O-C-C-N-C	
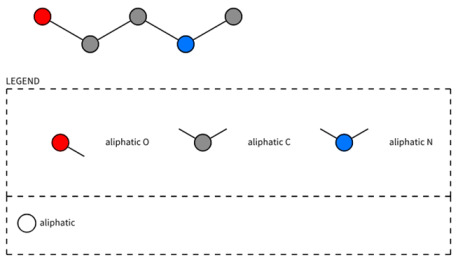
PubchemFP152	>=2 saturated or aromatic nitrogen-containing ring size 5	
PubchemFP797	CC1CC(C)CCC1	
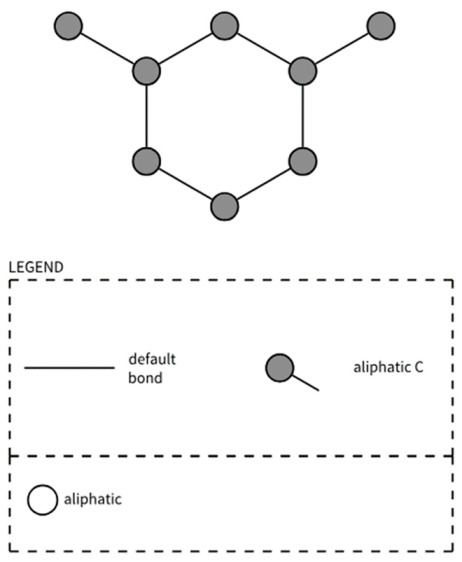
PubchemFP699	O-C-C-C-C-C(C)-C	
